# Preclinical Evaluation of a Pilocarpine–(R)-Lipoic Acid Eye Drop for Presbyopia

**DOI:** 10.1167/tvst.14.11.17

**Published:** 2025-11-14

**Authors:** Emily Robb, Korawin Triyasakorn, Jason Christidhis, Joshua Buffington, Marina L. Mamarian, Mahesh Kandula, Subbu Apparsundaram, John M. York

**Affiliations:** 1 Rutgers Institute for Pharmaceutical Industry Fellowships, Ernest Mario School of Pharmacy, Rutgers University, Piscataway, NJ, USA; 2 Akita Biomedical, Fallbrook, CA, USA; 3 Cellix Biosciences, Inc., Newark, NJ, USA; 4 Cellix Bio Private Limited, Hyderabad, India; 5 Institute for the Global Entrepreneur at the Jacobs School of Engineering and Rady School of Management, University of California, San Diego, CA, USA; 6 Burnett School of Medicine, Texas Christian University, Fort Worth, TX, USA

**Keywords:** antioxidant, eye drops, lipoic acid, ocular, pilocarpine, preclinical studies, presbyopia

## Abstract

**Purpose:**

Presbyopia is a progressive, age-related loss of near vision. Although current therapies offer symptomatic relief, they fail to target the underlying pathology. These studies investigated a novel dual-mechanism eye drop, CLX-162 (pilocarpine lipoate salt), focusing on three key characteristics: (1) tolerability, (2) pharmacokinetics and ocular tissue penetration, and (3) chemical stability within a dual-chamber delivery system.

**Methods:**

Tolerability and pharmacokinetic studies involved administering CLX-162 and lipoic acid choline ester (LACE) ophthalmic formulations to New Zealand White rabbits. Investigators assessed ocular tolerability using the Draize scoring system and evaluated pharmacokinetics by collecting and analyzing ocular tissues. A third study evaluated CLX-162 stability by storing it in a dual-chamber system under varying conditions and analyzing the drug substance and reconstituted product.

**Results:**

CLX-162 demonstrated superior ocular tolerability compared to LACE, with no corneal, iridial, or conjunctival effects observed. It induced transient pupillary constriction, whereas LACE caused mild redness and discharge. Pharmacokinetic analysis showed that CLX-162 achieved significantly higher and longer lasting (R)-lipoic acid levels in the aqueous humor and lens than LACE. Pilocarpine remained detectable for up to 8 hours. Stability studies confirmed that CLX-162 retained potency for 6 months in the dual-chamber container, with pilocarpine and (R)-lipoic acid levels within 95% to 100%. After reconstitution, it remained stable for 21 days.

**Conclusions:**

These preclinical studies demonstrated the stability, penetrability, and safety of CLX-162 dispensed in a dual-chamber, supporting progression to clinical trials.

**Translational Relevance:**

The dual-mechanism design of CLX-162 addresses the oxidative stress-driven lens changes underlying presbyopia, bridging preclinical findings to future patient care.

## Introduction

Presbyopia is an age-related decline in near vision, typically noticeable after the age of 40. This dynamic promotes disulfide cross-linking and decreases lens elasticity.^1^^,^^2^ If left untreated, this reduction in lens antioxidant levels may further exacerbate oxidative stress, leading to lens opacification and cataracts.^3^^,^^4^ U.S. Food and Drug Administration (FDA)-approved treatments for presbyopia include topical pilocarpine hydrochloride. Pilocarpine activates muscarinic receptors in the iris sphincter and ciliary muscles, inducing miosis to improve near vision.^5^^,^^6^ Emerging therapies include aceclidine, Brimochol (carbachol plus brimonidine), and MR-141 (pilocarpine plus phentolamine).^5^^,^^6^ Like pilocarpine, aceclidine also targets muscarinic receptors. In contrast, brimonidine and phentolamine are α-adrenoceptor agonists and antagonists, respectively. Although generally well tolerated, pilocarpine has a short duration of action (6–8 hours) due to its rapid metabolism and clearance, necessitating frequent reapplication.^5^ The adverse effects of pilocarpine include headache and eye redness.^5^^,^^6^ Surgical interventions, including lens replacement, offer longer term solutions but may impair binocular vision, are costly, and carry postoperative risks.^1^^,^^5^ These limitations underscore the need for non-invasive therapies that target the underlying biochemical mechanisms of presbyopia.

Lipoic acid, a naturally occurring antioxidant, may increase near vison in presbyopes by reducing disulfide cross-linking and improving lens elasticity. However, poor stability and limited corneal penetration limit its clinical use.^2^^,^^7^^,^^8^ A stabilized analog, lipoic acid choline ester (LACE), hydrolyzes into lipoic acid and subsequently into dihydrolipoic acid (DHLA). DHLA is an active agent that rejuvenates lens antioxidant levels and reduces disulfide cross-linking of lens proteins, thus restoring lens elasticity.^1^^,^^9^ Phase 1/2a trials of LACE have yielded mixed results, as one reported sustained near-vision improvement, and the other found no dose-dependent effect of LACE.^1^^,^^10^

**Figure 1.**
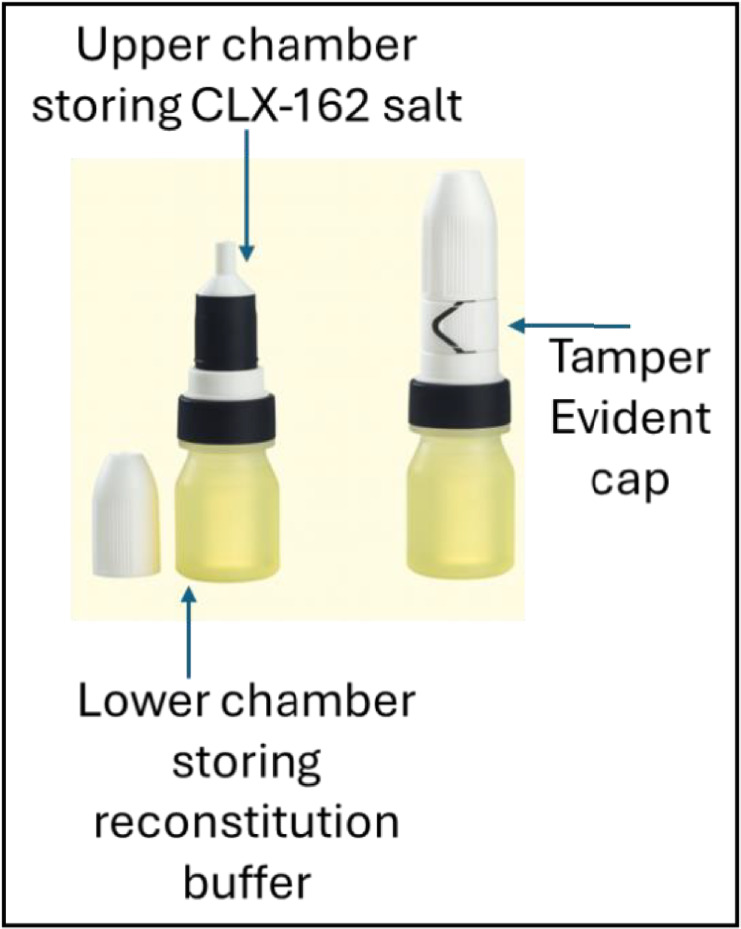
Dual-chamber bottle for CLX-162. The upper chamber houses the CLX-162 salt, and the lower chamber contains the sterile reconstitution buffer. A tamper-evident cap ensures sterility and stability of the product prior to activation. Upon reconstitution, the system facilitates accurate dosing and minimizes handling steps, thereby reducing the risk of contamination and improving ease of use in clinical settings. (Adapted from https://tekni-plex.com.)

CLX-162, a formulation of pilocarpine–(R)-lipoic acid salt, was developed to address both the symptomatic and biochemical aspects of presbyopia. Pilocarpine induces miosis, temporarily improving near vision.^7^ In parallel, (R)-lipoic acid has demonstrated preclinical activity in reducing oxidative stress and disulfide bond accumulation in lens proteins—processes implicated in loss of lens elasticity.^2^ Given these complementary mechanisms, CLX-162 may offer both immediate and sustained effects on visual function. However, the clinical application of (R)-lipoic acid has historically been limited by poor stability, rapid degradation, and inadequate corneal penetration, which may have contributed to inconsistent efficacy.^2^^,^^11^ To address these limitations, CLX-162 includes a dual-chamber container that stores the drug substance and reconstitution buffer separately (Fig. 1). This design aims to enhance product stability and maintain bioavailability during storage. Investigating whether CLX-162 overcomes these limitations was a key objective of the present studies.

Given the existing gaps in presbyopia management and the potential for overcoming these with novel CLX-162, this paper aims to address three critical research questions: (1) What is the tolerability of CLX-162? (2) What are the pharmacokinetics of CLX-162, particularly concerning (R)-lipoic acid tissue penetration and concentration in ocular tissues? and (3) What is the stability profile of CLX-162 when stored in a dual-chamber delivery system? This paper first describes the methodology for the three preclinical studies that address each research question. Next, it provides the key findings from each study. Finally, this paper assesses the translational relevance of these results.

## Methods

This research consisted of three studies. The tolerability and pharmacokinetic investigations were animal studies involving male New Zealand White rabbits, and the third was an in vitro stability study in a dual-chamber container system.

### Regulatory and Animal Models for Preclinical Tolerability and Pharmacokinetics

The preclinical tolerability and pharmacokinetics animal studies complied with the regulations established by the Institutional Animal Ethics Committee and followed the guidelines of the ARVO Statement for the Use of Animals in Ophthalmic and Vision Research. Animal care and experimental procedures adhered to the standards set by the Committee for the Purpose of Control and Supervision of Experiments on Animals for Laboratory Animal Facilities in India, in compliance with the Prevention of Cruelty to Animals Act (1960).

### Study Drugs

Drug substances included CLX-162 and LACE, both supplied by Suven Life Sciences Ltd. (Hyderabad, India). The CLX-162 formulation consisted of 29.4 mg/mL pilocarpine–(R)-lipoic acid, 27 mg/mL glycerin, 5 mg/mL L-alanine, and water for injection. The LACE formulation consisted of 30 mg/mL (R)-lipoic acid choline ester, 27 mg/mL glycerin, 5 mg/mL L-alanine, and water for injection.

### Tolerability Study

This study included four naïve male New Zealand White rabbits (4–5 months old; 2.1–2.4 kg) from Liveon Biolabs Pvt. Ltd. (Karnataka, India). Three rabbits received 50 µL of LACE (3% w/v) topically in the left eye and 50 µL of CLX-162 (2.94% w/v) in the right eye for 3 consecutive days. The fourth rabbit served as an untreated control.

Investigators assessed ocular damage/irritation using the Draize^12^ scoring system (Table 1) by examining the cornea, iris, and conjunctiva at 1, 4, 8, and 24 hours after the first application and at 1, 4, and 24 hours following subsequent doses. The scoring system included a maximum possible score of 80 for the cornea, 10 for the iris, and 20 for the conjunctiva.

**Table 1.** Draize Scoring System Using Ocular Tissue for the Evaluation of Tolerance

Ocular Tissue	Description	Score
I. Cornea	A. Opacity: degree of density	
	Scattered or diffuse areas of opacity (other than slight dulling of normal luster); details of iris clearly visible	1
	Easily discernible translucent area; details of iris clearly visible	2
	Opalescent areas; no details of iris visible; size of pupil barely discernible	3
	Opaque cornea; iris not discernible through the opacity	4
	B. Area of cornea involved	
	>0 to ≤1/4	1
	>1/4 to <1/2	2
	>1/2 to <3/4	3
	>3/4 to ≤ whole area	4
	Final score calculation: (A × B × 5)	×/80
II. Iris	A. Values	
	Folds have normal congestion, swelling, circumcorneal injection (any one or all of these or a combination of any thereof); iris still reacting to light (sluggish reaction is positive)	1
	No reaction to light; hemorrhage; gross destruction (any one or all)	2
	Final score calculation: (A × 5)	×/10
III. Conjunctivae	A. Redness (palpebral conjunctivae only)	
	Vessels definitely injected above normal	1
	More diffuse; deeper crimson red; individual vessels not easily discernible	2
	Diffuse beefy red	3
	B. Chemosis	
	Any swelling above normal (includes nictitating membrane)	1
	Obvious swelling, with partial eversion of lids	2
	Swelling, with lids about half closed	3
	Swelling, with lids more than half closed	4
	C. Discharge	
	Any amount different from normal (does not include small amounts observed in the inner canthus of normal animals)	1
	Discharge with moistening of the lids and hairs just adjacent to the lids	2
	Discharge with moistening of the lids and considerable area around the eye	3
	Final score calculation: (A + B + C) × 2	×/20

Investigators graded corneal opacity and scored the affected area of the cornea. The final corneal score resulted from the following formula: opacity × area × 5, where area quantified the extent of the affected region. Assessment of the iris involved evaluating the light-reactive iris (sluggish response positive), with a final iris score calculated as value × 5. Finally, for the conjunctiva, they evaluated redness, chemosis, and discharge separately and determined the final conjunctival score using the formula (redness + chemosis + discharge) × 2. A score of 0 in any parameter indicated no abnormalities observed. Investigators noted that no other ocular effects were present. Clinical observations included monitoring body weight (recorded on Day 1 before dosing and daily until the last observation) and feed consumption (recorded daily until the last day of observation).

### Ocular Pharmacokinetic Study

This study included 30 naïve male New Zealand White rabbits (4–5 months old; 1.9–2.6 kg) from Liveon Biolabs Pvt. Ltd. The rabbits received 50 µL of either topical CLX-162 (2.94% w/v) or LACE (3% w/v) formulation (total volume of 100 µL). Following application, investigators terminated the rabbits at predetermined time points to collect lens, cornea, and iris samples. The study treated 15 rabbits with CLX-162 and 15 with LACE, collecting aqueous humor, cornea, iris, and lens samples from three rabbits per time point per formulation at 0.5, 1, 2, 4, and 8 hours post-application. Investigators quantified the aqueous humor samples for (R)-lipoic acid in both groups and pilocarpine in the CLX-162 group. They also quantified the lens samples for (R)-lipoic acid in both treatment groups and the iris samples for pilocarpine in the CLX-162 treatment group. One minute before sample collection, investigators anesthetized the corneas with 2% lignocaine (∼30 µL) and collected ∼100 µL of aqueous humor from each eye using a 30-gauge needle. They immediately mixed samples with 2 µL of stabilizer (100-mM phenylmethyl sulfonyl fluoride in dimethyl sulfoxide:glacial acetic acid, 50:50). After euthanasia, investigators removed the eyeballs to collect the cornea, iris, and lens. The investigators then immediately snap-froze the samples in liquid nitrogen, placed them on dry ice, and stored them at –70°C until analysis.

Researchers analyzed samples using liquid chromatography–tandem mass spectrometry (LC-MS/MS) with a fit-for-purpose method to quantify pilocarpine accurately and (R)-lipoic acid in rabbit ocular tissues and fluids. Calibration and quality control samples ensured precise measurement in the aqueous humor, iris, and lens. Data analysis used Analyst Version 1.6.3 (SCIEX, Marlborough, MA), with descriptive statistics (mean, standard deviation) calculated for analyte concentrations. Investigators determined key pharmacokinetic parameters (*C_max_*, *T_max_*, and *AUC_last_*) for both compounds. Investigators constructed a composite pharmacokinetic profile by averaging concentrations from different animals at each time point. Because each animal contributed data to only one time point, investigators did not calculate the standard deviation for the pharmacokinetic parameters.

### CLX-162 Stability Study in Dual-Chamber Containers

To determine the shelf-life and stability of CLX-162 before and after reconstitution, investigators placed 170 mg of CLX-162 in the upper chamber of a dual-chambered container (TekniPlex LF of America, Boca Raton, FL). They stored 10 mL of proprietary reconstitution buffer in the lower chamber. Investigators then stored the containers in temperature- and humidity-controlled stability chambers under the following conditions: (1) 2°C to 8°C, (2) 25°C with 40% relative humidity, (3) 30°C with 65% relative humidity, and (4) 40°C with 25% relative humidity, for durations of 1, 2, 3, and 6 months.

At each specified time point, investigators removed the dual-chambered containers from the stability chambers and inspected CLX-162 for any changes in physical appearance. Investigators then released the buffer from the lower chamber to reconstitute CLX-162, yielding a homogeneous solution. On the day of reconstitution (Day 1), investigators recorded the physical properties, including clarity, color, and evidence of precipitation, and collected an aliquot of the solution for HPLC analysis (Supplementary Tables S1 and S2). Investigators stored the reconstituted containers in the stability chamber for 21 days. On Day 21 post-reconstitution, investigators removed the containers, inspected the physical appearance, and collected another aliquot for high-performance liquid chromatography (HPLC) analysis. Investigators analyzed pilocarpine–(R)-lipoic acid, EDTA, pilocarpic acid, isopilocarpine, and benzalkonium chloride using HPLC.

## Results

### Tolerability Study

In all treated rabbits, researchers observed no clinical signs related to the systemic effects of the treatment. Body weight changed minimally, ranging from –4.9% to –0.5% from Days 1 through 4. With regard to ocular tolerability, based on the Draize scoring system,^12^ the rabbits tolerated the CLX-162 formulation better than the LACE formulation (Table 2). Researchers noted no corneal, iridial, or conjunctival effects in the eye treated with CLX-162 throughout the study. Individual ocular irritation scores at 1, 4, and 8 hours post-treatment in the CLX-162–treated eye remained normal on Days 1, 2, and 3, with no observed corneal, iridial, or conjunctival effects. CLX-162 caused pupillary constriction after each instillation, which subsided within 1 hour. LACE caused no corneal or iridial damage but minimal redness in all three rabbits. All animals developed ocular discharge in the LACE-treated eye within an hour post-treatment on the first day, but the discharge subsided by Day 2 in all rabbits except one. Researchers observed discharge and minimal redness in the LACE-treated eye of two rabbits on Day 1 and one rabbit on Day 2.

**Table 2.** Individual Total Scores and Group Mean Scores for Ocular Irritation

		Rabbit #1		Rabbit #2		Rabbit #3		Group Mean Score	
	Time	Left Eye	Right Eye	Left Eye	Right Eye	Left Eye	Right Eye	Left Eye	Right Eye
Study Day	Post-Treatment (h)	LACE	CLX-162	LACE	CLX-162	LACE	CLX-162	LACE	CLX-162
1	1	0	0	2	0	2	0	1.33	0
	4	2	0	0	0	2	0	1.33	0
	8	2	0	0	0	0	0	0.67	0
	24	2	0	0	0	0	0	0.67	0
2	1	0	0	4	0	0	0	1.33	0
	4	0	0	2	0	0	0	0.67	0
	24	0	0	0	0	0	0	0	0
3	1	0	0	0	0	0	0	0	0
	4	0	0	2	0	0	0	0.67	0
	24	0	0	0	0	0	0	0	0

### Ocular Pharmacokinetic Study

**Figure 2.**
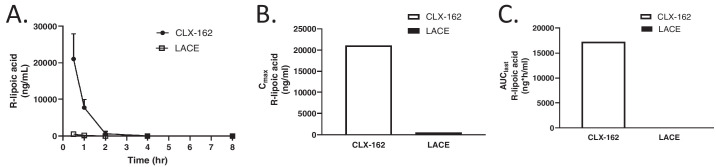
(R)-lipoic acid concentration in the rabbit aqueous humor following treatment with CLX-162 and LACE formulations. (**A**) (R)-lipoic concentration in the aqueous humor at different time points. Data are presented as mean ± SD (*n* = 3 rabbits/time point). (**B**) *C_max_* of (R)-lipoic acid. (**C**) AUC of (R)-lipoic acid.

Figure 2 illustrates the pharmacokinetics of (R)-lipoic acid in aqueous humor after applying CLX-162 and LACE formulations. In the aqueous humor, (R)-lipoic acid reached its maximum concentration at 0.5 hour post-treatment and then gradually decreased over the subsequent 8 hours. CLX-162 treatment maintained detectable levels of (R)-lipoic acid for up to 4 hours, whereas LACE treatment resulted in undetectable levels by 2 hours post-treatment. CLX-162 treatment produced a maximal lipoic acid concentration approximately 40 times higher than LACE treatment, with *C_max_* values of 21,067 ng/mL and 524 ng/mL, respectively.

**Figure 3.**
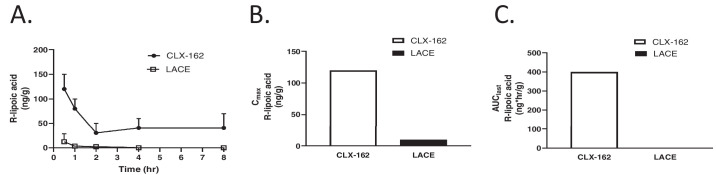
(R)-lipoic acid concentration in the rabbit lens following treatment with CLX-162 and LACE formulations. (**A**) (R)-lipoic acid concentration in the lens at different time points. Data are presented as mean ± SD (*n* = 3 rabbits/time point). (**B**) *C_max_* of (R)-lipoic acid. (**C**) AUC of (R)-lipoic acid.

Figure 3 depicts the pharmacokinetics of (R)-lipoic acid in the lens after administration of CLX-162 and LACE formulations. In the lens, (R)-lipoic acid reached its maximal concentration at 0.5 hour post-treatment and gradually decreased. CLX-162 treatment sustained detectable levels of lipoic acid until 8 hours, whereas LACE treatment led to undetectable levels of (R)-lipoic acid by 4 hours. CLX-162 treatment resulted in a maximal (R)-lipoic acid concentration approximately 10 times higher than that for LACE treatment, with *C_max_* values of 120 ng/g and 10 ng/g following CLX-162 and LACE treatment, respectively. Notably, (R)-lipoic acid concentration in the lens remained relatively stable between 4 and 8 hours following CLX-162 treatment. The *AUC_last_* values for (R)-lipoic acid in the CLX-162 group were 17.271 µg·h/mL in the aqueous humor and 0.40 µg·h/g in the lens, respectively. Investigators could not calculate the *AUC_last_* values for the LACE group.

**Figure 4.**
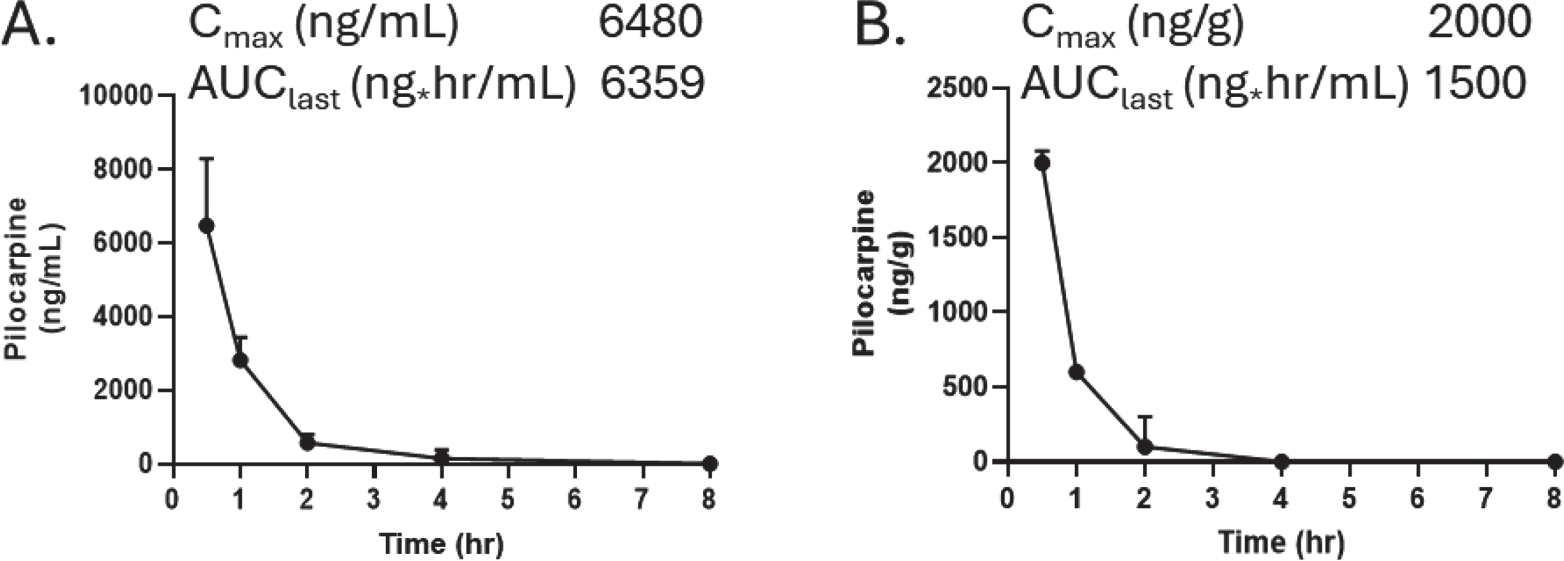
(**A, B**) Pilocarpine concentrations in the aqueous humor (**A**) and iris (**B**) from rabbits treated with CLX-162 formulation.

Figure 4 displays the pharmacokinetics of pilocarpine in the aqueous humor and lens after applying the CLX-162 formulation. Pilocarpine reached its maximum concentration in the aqueous humor at 0.5 hour post-treatment, with a *C_max_* of 6.48 µg/mL, and then gradually decreased during the subsequent 8 hours. CLX-162 treatment maintained detectable levels of pilocarpine for 4 hours. Pilocarpine reached a *C_max_* of 1.962 µg/g in the iris at 0.5 hour post-treatment and remained detectable until 8 hours. The *AUC_last_* values for pilocarpine were 6.36 µg·h/mL in the aqueous humor and 1.50 µg·h/g in the iris, respectively.

### CLX-162 Stability Study in Dual-Chamber Containers

**Figure 5.**
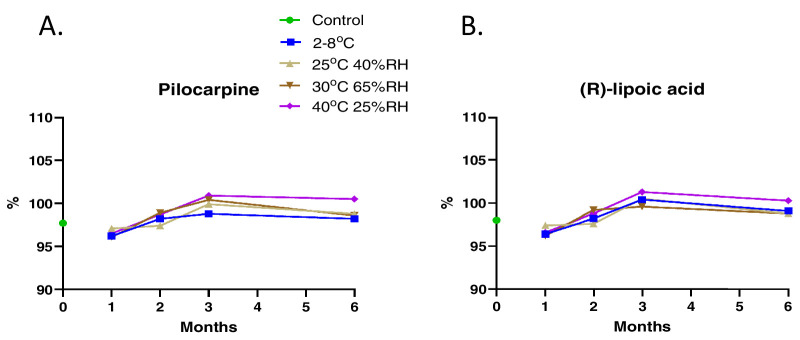
Stability testing of CLX-162 at 1, 2, 3, and 6 months. The upper chamber of the dual-chamber container stored CLX-162 while the container was kept under different conditions at various time points. (**A**) The percentage of pilocarpine compared to the control under different conditions. (**B**) The percentage of (R)-lipoic acid compared to the control at various time points.

**Figure 6.**
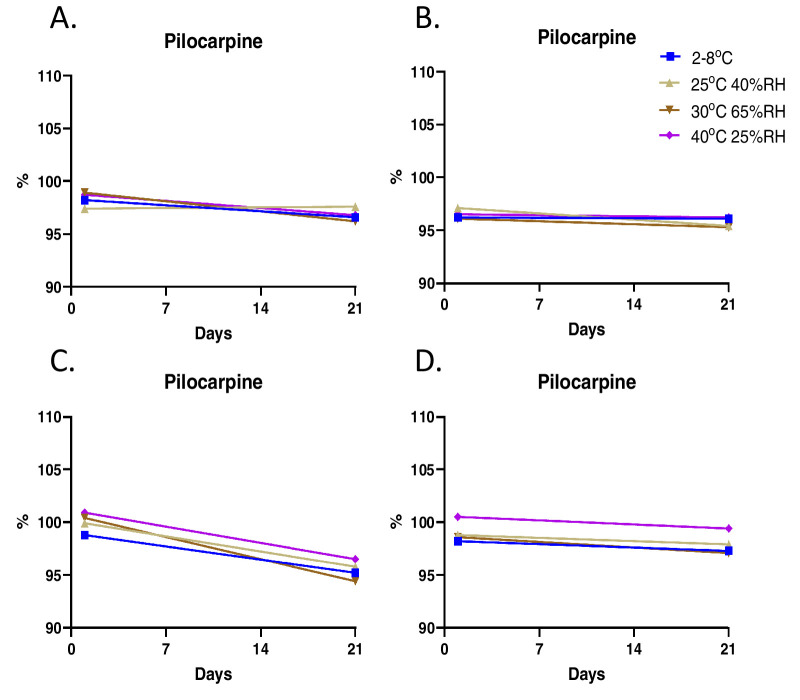
Stability testing of CLX-162 reconstituted in the buffer on Day 1 and Day 21 after reconstitution at various time points. The percentage of pilocarpine is compared to control CLX-162. (**A–D**) Investigators reconstituted CLX-162 stored in the upper chamber of the dual-chamber container under various conditions at 1 month (**A**), 2 months (**B**), 3 months (**C**), and 6 months (**D**) and determined the percentage of pilocarpine compared to the control.

**Figure 7.**
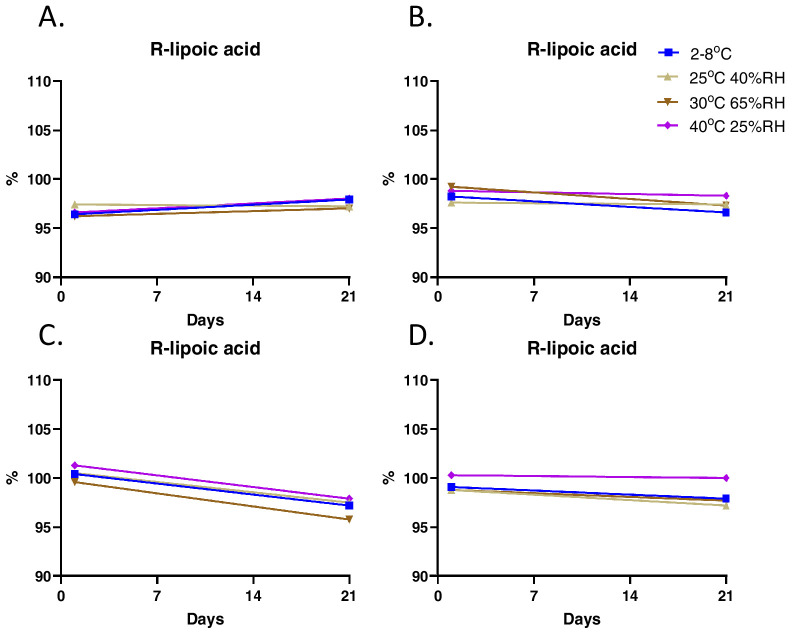
Stability testing of CLX-162 reconstituted in the buffer on Day 1 and Day 21 after reconstitution at various time points. The percentage of (R)-lipoic acid is compared to control CLX-162. (**A–D**) Investigators reconstituted CLX-162 stored in the upper chamber of the dual-chamber container under various conditions at 1 month (**A**), 2 months (**B**), 3 months (**C**), and 6 months (**D**) and determined the percentage of (R)-lipoic acid compared to the control.

Initial studies on CLX-162 formulations revealed that (R)-lipoic acid decreased to less than 90% within 60 days of formulation when stored at ambient temperature. To improve the long-term stability of CLX-162, investigators selected a dual-chamber container designed by TekniPlex LF of America (Wayne, PA), as it stores CLX-162 with excipients and the reconstitution buffer in separate chambers until mixed, preserving the integrity and efficacy of CLX-162. HPLC analysis of CLX-162 revealed the presence of pilocarpine, pilocarpine acid, and isopilocarpic acid as related impurities generated during manufacturing. All impurities remained within the limits proposed for the pilocarpine hydrochloride United States Pharmacopeia (USP) monograph (Supplementary Tables S3–S6). Stability studies of the CLX-162 drug substance revealed that pilocarpine and (R)-lipoic acid levels remained stable at 95% to 100% across all tested conditions (Fig. 5). Reconstituted CLX-162 also demonstrated stability, with pilocarpine (Fig. 6) and (R)-lipoic acid levels remaining within 95% to 100% at the 21-day time point (Fig. 7).

## Discussion

This preclinical research evaluated the tolerability, ocular pharmacokinetics, and chemical stability of CLX-162, a novel pilocarpine–(R)-lipoic acid ophthalmic formulation, using a New Zealand White rabbit model. This work demonstrated that the animals tolerated CLX-162 well. CLX-162 exhibited favorable ocular pharmacokinetic properties, showing detectable concentrations in the aqueous humor and lens for up to 8 hours post-administration. The 6-month accelerated stability testing revealed that CLX-162 retained chemical stability within a dual-chamber container for at least 6 months. These findings support the continued development of CLX-162 as a potential therapeutic for presbyopia.

This preclinical study utilized a New Zealand White rabbit model and the standardized accelerated stability testing established by the International Council for Harmonisation of Technical Requirements for Pharmaceuticals for Human Use (ICH). Researchers have accepted the rabbit model as a standard in ophthalmology research.^13^ Rabbits have provided a good model for assessing the safety and efficacy of innovative approaches and drug development due to their anatomical similarities to humans, including eye size, internal structures, optical system, and conjunctival cavity volume. Many researchers have utilized this model to study diverse ocular pathologies and therapeutics.^14^ For example, Detorakis et al.^15^ evaluated shear-wave elastography in rabbits’ eyes following the administration of atropine and pilocarpine ophthalmic drops, and Szumny et al.^16^ investigated the effect of plant-derived compounds on intraocular pressure in this model. These studies have offered support the validity of this animal model for evaluating the tolerability and ocular penetration of novel formulations.

Although presbyopia affects daily activities such as reading, it also represents a significant precursor to cataracts, a debilitating condition requiring surgical intervention.^6^ Current treatments for presbyopia primarily include corrective lenses as well as topical pilocarpine.^5^ These interventions provide symptomatic relief but do not address the underlying pathophysiology of presbyopia. Lens antioxidant levels progressively decline due to increased oxidative stress starting after the age of 40.^3^^,^^4^^,^^17^ Initially, oxidative stress increases lens stiffness by creating disulfide cross-links in lens proteins. These cross-links reduce lens elasticity, impairing accommodation and the ability to focus on near objects.^18^^,^^19^ With a continued decline in antioxidant levels, particularly in individuals 70 years and older, oxidative stress further intensifies.^20^ In this continuum, protein aggregation occurs alongside protein cross-linking, resulting in insoluble proteins and lens opacification, ultimately forming cataracts.^3^ Antioxidants in the lens cortex exhibit a moderate change, but the decline is more significant in the lens nucleus.^21^ Over time, the progressive oxidative stress contributes to presbyopia and early cataractogenesis, establishing the lens as a compelling target for antioxidant therapy.

Topical pilocarpine induces temporary miosis, enhancing near vision by causing pupillary constriction and ciliary muscle contraction via muscarinic receptor activation, which lasts for only 6 to 8 hours, necessitating frequent dosing to achieve sustained benefits.^6^ Additionally, pilocarpine use is associated with a range of adverse effects, including headaches, brow aches resulting from sustained contraction of the ciliary muscles,^22^ and impaired night vision due to sustained miosis.^5^ Another therapeutic alternative is 1.75% aceclidine (VIZZ), a recently FDA-approved muscarinic receptor agonist. The 1.75% aceclidine demonstrated rapid effects, with significant improvement in near vision lasting up to 10 hours in its phase 3 trial (LENZ Therapeutics, Solona Beach, CA). Most side effects were mild, including eye irritation and headaches.^23^ These agents offer only transient correction and do not target the biochemical drivers of presbyopia progression.

Lipoic acid is a natural antioxidant present in the lens of young adults that decreases with age to undetectable levels in older adults.^24^ Thus, lipoic acid supplementation has emerged as a potential agent to treat presbyopia. Lipoic acid, in its reduced form, DHLA, can directly neutralize reactive oxygen species; regenerate lens antioxidants, including glutathione, vitamin C, and vitamin E^25^; and reduce or break disulfide cross-links in proteins due to its strong reducing (thiol) properties.^26^ Garner and Garner^2^ demonstrated that lipoic acid reduces the accumulation of lens crystallin proteins by cleaving disulfide bonds in murine models. Although (R)-lipoic acid is a biologically active isomer of lipoic acid, its stability and ocular permeability challenges limit its clinical use. (R)-lipoic acid is highly unstable in aqueous solutions, especially at physiological pH (∼7.4).^27^ It readily undergoes oxidation, forming disulfide dimers or degrading into inactive products. (R)-lipoic acid degrades rapidly under light and at elevated temperatures, limiting its shelf life and complicating its formulation for ophthalmic use.^7^ Due to its physicochemical properties, (R)-lipoic acid exhibits poor corneal permeability, resulting in subtherapeutic concentrations in the aqueous humor and lens when administered topically.^7^

In phase 1/2 studies, LACE (1.5%), designed to enhance (R)-lipoic acid permeability into ocular tissues when applied twice daily for 90 days, demonstrated improvement in distance-corrected near visual acuity, with the improvement in near vision persisting for up to 7 months after treatment cessation.^1^ However, the dose-ranging phase 2b study failed to achieve a statistically significant dose–response effect, resulting in termination of the study (NCT04806503).^10^ In the current pharmacokinetic study, CLX-162 achieved greater levels of (R)-lipoic acid in the lens compared to LACE. This finding may be partly due to the dosage formulation and partly due to the presence of pilocarpine in CLX-162. Pilocarpine, due to its pupillary constriction and ciliary muscle contraction actions, may alter anterior chamber diffusion dynamics, improving the diffusion of (R)-lipoic acid to the lens.^28^ Moreover, pilocarpine may increase the aqueous humor outflow by stretching the trabecular meshwork, which may enhance the distribution and ocular exposure of (R)-lipoic acid.^29^ Although this study did not evaluate direct preclinical evidence, the proposed mechanisms suggest that pilocarpine and (R)-lipoic acid have a pharmacodynamic synergy that warrants further exploration.

Traditional multi-dose ophthalmic products often require higher concentrations of preservatives to maintain sterility, leading to ocular irritation and toxicity when used for prolonged periods.^30^ The dual-chamber container presents an advanced packaging solution, providing a step forward in ophthalmic drug delivery, ensuring efficacy, patient safety, and ease of use. CLX-162 drug substance stored in a dual-chamber container showed good stability during 6 months of storage, with pilocarpine and (R)-lipoic acid levels conforming to specifications. By keeping the drug substance and the reconstitution buffer separate until reconstitution, the formulation maintains optimal pH, osmolarity, and drug solubility, resulting in improved absorption and therapeutic outcomes until the time of application.

Like most preclinical studies, this work has important limitations. First, although the New Zealand White rabbit is a widely used model in ophthalmic research and provides reliable data on ocular pharmacokinetics and tolerability, it does not fully replicate the age-related lens changes that drive presbyopia in humans.^13^^,^^14^^,^^31^ As such, although our results support the translational potential of CLX-162, additional testing in non-human primates, which more closely mimic the anatomy and physiology of the human accommodative system, will be critical for confirming relevance.^32^ Second, the relatively small sample size, though sufficient for exploratory pharmacokinetic and tolerability endpoints, limited the statistical power and generalizability. Larger preclinical cohorts and eventual clinical studies will be necessary to validate these findings with greater confidence. Third, investigators conducted the preclinical studies using 1.5% CLX-162 but could use additional strengths in future studies. Finally, although pilocarpine is effective in transiently improving near vision by contracting the ciliary muscle and constricting the pupil, there is a theoretical risk that repeated use could bias the accommodative apparatus toward a near-focus state. However, recent evidence suggests that low pilocarpine concentrations produce only short-acting and reversible effects,^22^ and in our formulation, pilocarpine may also enhance anterior chamber fluid dynamics to facilitate lens penetration of (R)-lipoic acid.^28^^,^^29^ Nonetheless, the long-term impact of pilocarpine on accommodative flexibility requires further study. Taken together, these limitations underscore the need for future investigations that (1) employ non-human primate models, (2) expand sample sizes, (3) vary strengths, and (4) characterize the pharmacodynamic role of pilocarpine in combination therapy. These steps will be essential for advancing CLX-162 toward clinical application in presbyopia.

With regard to the stability of the reconstituted CLX-162, although not clinically meaningful, Figures 6C and 7C show a clear decline with time in percentage of pilocarpine and lipoic acid 3 months after but not 6 months after the reconstitution. This difference can be due to the sample size and experimental variation considerations.

In conclusion, this preclinical research demonstrated that CLX-162 dispensed from a dual-chamber bottle achieves excellent penetration into the aqueous humor and lens, maintains an extended shelf life in its dual-chamber storage container, and presents a favorable safety profile consistent with the established characteristics of its pharmacologic components. CLX-162 has the potential to treat both the symptomatic and underlying causes of presbyopia. Future clinical trials will determine if the safety and efficacy of CLX-162 translate in human subjects, paving the way for a potential breakthrough in managing presbyopia.

## Supplementary Material

Supplement 1
