# Supplementary material for: Preclinical Evaluation of a Pilocarpine–(R)-Lipoic Acid Eye Drop for Presbyopia
Source: Transl Vis Sci Technol. 2025 Nov 14;14(11):17. doi: 10.1167/tvst.14.11.17 (PMC12629135; doi:10.1167/tvst.14.11.17)
Supplement: Supplement 1 [file tvst-14-11-17_s001.pdf]

Supplementary Materials

S. No	Test	Specification	Result
**1**	Physical Appearance	Yellow to pale yellow color crystalline powder	Pale yellow-colored crystalline powder
**2**	Solubility in water	Freely soluble	Freely soluble
**3**	Identification by		
	IR	Should confirm	Confirms
	3H and 13C-NMR	Should confirm	Confirms
	ESI-Mass	Should confirm	Confirms
**4**	Loss on drying at 40 C $\pm$ 2 C (3 hours under vacuum at 600-650 mm/Hg), % w/w	Not more than 3.0	0.24
**5**	Residue on ignition, % w/w	Not more than 0.10	0.06
**6**	Differential Scanning Colorimetry (2 C/min)	69-76	72.33
**7**	Pilocarpine and (R)-lipoic acid content by HPLC (on dried basis), % w/w		
	Pilocarpine	49.22-51.33	50.59
	(R)-Lipoic acid	48.77-50.77	49.93
**8**	Related substances by HPLC		
	Isopilocarpine	Not more than 1.0%	0.7
	Pilocarpic acid	Not more than 0.5%	Not detected
	Isopilocarpic acid	Not more than 0.15%	Not detected
	Any unspecified impurity	Not more than 0.10%	0.02
	Total impurities	Not more than 2.0%	0.13
**9**	Specific Optical Rotation	dextro 109 to 119	114.9
**10**	Residual solvents by GC-HS, ppm		
	Acetone	Not more than 5000	Not detected
	Ethyl acetate	Not more than 5000	817
	Cyclohexane	Not more than 3800	Not detected
	Toluene	Not more than 890	Not detected
**11**	Purity	HPLC purity	99.49

Supplementary Table S1: Specifications for CLX-162 used in the study.

S. No	Test	Specification	Result
**1**	Physical Appearance	10mL Natural LDPE Container with white reservoir and perforator closed with LDPE Cap	Confirms
**2**	Solution Appearance	Pale yellow colored solution	Confirms
**3**	pH	5.5 - 6.5	6.1
**4**	Osmolality	200 - 350 m Osmol/kg	278
**5**	Viscosity	10 - 20 cps	17.14
**6**	EDTA 1.3 mg/ml	80% - 120% of the labeled amount	99.3
**5**	Benzalkonium chloride 0.1 mg/ml	80% - 120% of the labeled amount	98
**6**	Pilocarpine content by HPLC	90% - 110% of the label claim	97.7
**7**	(R)-lipoic acid content by HPLC	90% - 110% of the label claim	98
**8**	Pilocarpine (R)-lipoate	90% - 110% of the label claim	97.9
**9**	The related substance of pilocarpine by HPLC		
	Pilocarpic acid	Not more than 3%	0.02%
	Isopilocarpine	Not more than 1.0%	0.10%
	Isopilocarpic acid	Not more than 0.3%	ND
	Any unspecified impurity	Not more than 1.0%	0.02%
	Total impurities	Not more than 5.0%	0.14%
**10**	Related substance of (R)-lipoic acid by HPLC		
	Impurity of (R)-lipoic acid	Not more than 1.0%	0.03%
	Any other impurity	Not more than 1.0%	0.02%
	Total impurities	Not more than 2.0%	0.06%

Supplementary Table S2: Specifications of CLX-162 reconstituted solution used in stability studies.

S. No	Test	Limits	Control	Month							
				1		2		3		6	
				D1	D21	D1	D21	D1	D21	D1	D21
**1**	Assay of EDTA (%)	80-120	99	100		98		100		99	
**2**	Assay of benzalkonium (%)	80-120	98	99		103		101		98	
**3**	Related substance of pilocarpine (%)										
	Pilocarpic acid	NMT 3.0	0.02	0.06	2.03	0.20	2.08	0.68	2.05	0.06	2.08
	Isopilocarpine	NMT 1.0	0.10	0.11	0.70	0.14	0.73	0.12	0.73	0.10	0.71
	Isopilocarpic acid	NMT 0.3	ND	ND	0.01	ND	ND	ND	ND	ND	0.01
	Any unspecified impurity	NMT 1.0	0.02	0,023	0.02	0.03	0.02	0.02	0.02	0.02	0.02
	Total impurities	NMT 5.0	0.14	0.19	2.76	0.37	2.84	0.21	2.79	0.18	2.82
**4**	Related substance of (R)-lipoic acid (%)										
	Impurity A of (R)-lipoic acid	NMT 1.0	0.03	0.05	0.03	ND	0.04	0.04	0.10	0.04	0.03
	Any other impurity	NMT 1.0	0.02	0.04	0.59	0.02	0.10	0.03	0.62	0.05	0.62
	Total impurities	NMT 2.0	0.06	0.12	1.07	0.04	1.02	0.10	1.15	0.12	1.10

Supplementary Table S3: Stability Study Report on CLX-162 Additives and Impurities at 2^0-8^0C.

S. No	Test	Limits	Control	Month							
				1		2		3		6	
				D1	D21	D1	D21	D1	D21	D1	D21
**1**	Assay of EDTA (%)	80-120	99	98		99		99		100	
**2**	Assay of benzalkonium (%)	80-120	98	99		100		100		98	
**3**	Related substance of pilocarpine (%)										
	Pilocarpic acid	NMT 3.0	0.02	0.04	2.01	0.20	2.06	0.06	2.07	0.04	2.08
	Isopilocarpine	NMT 1.0	0.10	0.10	0.70	0.15	0.72	0.12	0.73	0.10	0.73
	Isopilocarpic acid	NMT 0.3	ND	ND	0.01	ND	ND	ND	ND	ND	0.01
	Any unspecified impurity	NMT 1.0	0.02	0.04	0.02	0.03	0.02	0.02	0.02	0.02	0.02
	Total impurities	NMT 5.0	0.14	0.18	2.74	0.38	2.81	0.21	2.82	0.17	2.82
**4**	Related substance of (R)-lipoic acid (%)										
	Impurity A of (R)-lipoic acid	NMT 1.0	0.03	0.05	0.03	ND	0.05	0.04	0.04	0.04	0.02
	Any other impurity	NMT 1.0	0.02	0.03	0.57	0.02	0.55	0.04	0.61	0.05	0.62
	Total impurities	NMT 2.0	0.06	0.11	1.04	0.04	1.02	0.11	1.04	0.12	1.08

Supplementary Table S4: Stability Study Report on CLX-162 Additives and Impurities at 25^{0}C / 40% RH.

S. No	Test	Limits	Control	Month							
				1		2		3		6	
				D1	D21	D1	D21	D1	D21	D1	D21
**1**	Assay of EDTA (%)	80-120	99	95		99		101		99	
**2**	Assay of benzalkonium (%)	80-120	98	98		102		102		97	
**3**	Related substance of pilocarpine (%)										
	Pilocarpic acid	NMT 3.0	0.02	0.05	2.02	0.21	2.08	0.09	2.07	0.07	2.08
	Isopilocarpine	NMT 1.0	0.10	0.10	0.72	0.16	0.74	0.14	0.75	0.14	0.74
	Isopilocarpic acid	NMT 0.3	ND	ND	0.01	ND	ND	ND	0.02	ND	0.01
	Any unspecified impurity	NMT 1.0	0.02	0.02	0.02	0.03	0.02	0.02	0.02	0.02	0.02
	Total impurities	NMT 5.0	0.14	0.17	2.77	0.40	2.85	0.25	2.84	0.23	2.85
**4**	Related substance of (R)-lipoic acid (%)										
	Impurity A of (R)-lipoic acid	NMT 1.0	0.03	0.05	0.03	ND	0.03	0.02	0.03	0.04	0.03
	Any other impurity	NMT 1.0	0.02	0.04	0.59	0.02	0.55	0.03	0.61	0.05	0.61
	Total impurities	NMT 2.0	0.06	0.11	1.08	0.04	1.00	0.09	1.04	0.13	1.08

Supplementary Table S5: Stability Study Report on CLX-162 Additives and Impurities at 36^{0}C / 65% RH.

S. No	Test	Limits	Control	Month							
				1		2		3		6	
				D1	D21	D1	D21	D1	D21	D1	D21
**1**	Assay of EDTA (%)	80-120	99	97		99		102		101	
**2**	Assay of benzalkonium (%)	80-120	98	98		104		101		99	
**3**	Related substance of pilocarpine (%)										
	Pilocarpic acid	NMT 3.0	0.02	0.05	2.03	0.21	2.07	0.07	2.09	0.05	2.11
	Isopilocarpine	NMT 1.0	0.10	0.10	0.70	0.18	0.75	0.16	0.77	0.18	0.80
	Isopilocarpic acid	NMT 0.3	ND	ND	0.01	ND	ND	ND	ND	ND	0.02
	Any unspecified impurity	NMT 1.0	0.02	0.02	0.02	0.03	0.02	0.03	0.03	0.02	0.22
	Total impurities	NMT 5.0	0.14	0.17	2.76	0.42	2.85	0.25	2.89	0.25	2.94
**4**	Related substance of (R)-lipoic acid (%)										
	Impurity A of (R)-lipoic acid	NMT 1.0	0.03	0.05	0.03	ND	0.05	0.03	0.03	0.05	0.04
	Any other impurity	NMT 1.0	0.02	0.03	0.59	0.02	0.57	0.04	0.63	0.05	0.63
	Total impurities	NMT 2.0	0.06	0.11	1.06	0.05	1.04	0.09	1.12	0.14	1.12

Supplementary Table S6: Stability Study Report on CLX-162 Additives and Impurities at 40^0C / 25% RH.
